# The radiological and electrophysiological characteristics of Hirayama disease with proximal involvement: A retrospective study

**DOI:** 10.3389/fneur.2022.969484

**Published:** 2022-08-11

**Authors:** Hongwei Wang, Ye Tian, Jianwei Wu, Chi Sun, Cong Nie, Chaojun Zheng, Fei Zou, Xinlei Xia, Xiaosheng Ma, Feizhou Lyu, Jianyuan Jiang, Hongli Wang

**Affiliations:** ^1^Department of Orthopedics, Huashan Hospital, Fudan University, Shanghai, China; ^2^Spine Center Fudan University, Shanghai, China; ^3^Department of Orthopedics, Shanghai Fifth People's Hospital, Fudan University, Shanghai, China

**Keywords:** Hirayama disease, proximal, radiography, electromyography, amyotrophic lateral sclerosis, motor neuron disease

## Abstract

**Purpose:**

Hirayama disease (HD) has been largely believed to affect only distal muscles. However, the proximal upper extremities have been affected in some cases, which can be confused with motor neuron diseases.

**Methods:**

Baseline data, deep tendon reflex, Hoffmann sign, cervical curvature, sagittal Cobb angle, atrophied spinal cord, high signal intensity, loss of attachment, and affected muscles and segments on electromyography (EMG) were retrospectively obtained and compared between patients with HD with proximal involvement and patients with simple distal HD in one center from September 2007 to April 2022.

**Results:**

In this study, fifteen patients with proximal HD and 30 patients with simple distal HD were included. The proximal group had a larger proportion of patients with decreased biceps reflex, decreased triceps reflex, brisk or hyperactive knee reflex, positive Hoffmann sign, and cervical kyphosis. The curvatures of the upper part of the cervical spine (C2-4) were lost to a greater degree in the proximal group. More affected segments were observed on magnetic resonance imaging (MRI) and electromyography in the proximal group.

**Conclusion:**

The injured segments were longer and the upper curvature of the cervical spine was poorer in patients with HD with proximal involvement. These findings indicated that proximal involvement may indicate more serious HD.

## Introduction

Hirayama disease (HD) is characterized by unilateral or bilateral weakness and muscular atrophy in the upper extremities caused by cervical flexion injury (1). Adolescent men are more prone to HD following a growth spurt when the cervical spinal cord and spine differ in length (2). Hirayama disease has been historically believed to affect only the distal muscles in the upper extremities. However, proximal upper limb atrophy and weakness have been reported (3–5). Among patients with HD, 8.8% complained of muscular atrophy of the biceps brachii in a nationwide survey in Japan (6), and 3.3–13% of patients complained of atrophy of the deltoid or biceps in China (7, 8). These findings demonstrate that HD can affect the proximal muscles of the upper extremities.

Patients diagnosed with HD with proximal involvement typically complain of amyotrophy of the proximal and distal upper limbs and reduced quality of life. Some patients experience generalized unilateral or bilateral muscle atrophy of the upper extremities, which requires exclusion of a number of neurological diseases, i.e., motor neuron disease, prior to diagnosis of HD, to avoid the physical, psychological, and economic burden for misdiagnosis. Therefore, strict criteria for the diagnosis of HD with proximal involvement are needed to prevent misdiagnosis and potential negative outcomes. Some criteria have been defined (9, 10), but controlled studies and synthesis of results across studies are lacking.

Clinical-led guidelines and new diagnostic criteria have indicated that the diagnosis of HD requires a three-dimensional diagnostic framework, including clinical manifestations, imaging, and electrophysiological examinations (1, 11). These criteria de-emphasized distal muscle atrophy and indicated that clinical manifestations should receive less consideration with regard to the diagnosis of HD. In this study, we analyzed the radiological and electrophysiological characteristics of HD with proximal involvement and simple distal HD retrospectively to determine the specific characteristics of HD with proximal involvement. It helps not only in avoiding the misdiagnoses of such patients as other diseases, but enriches the concept of HD by including patients with different clinical manifestations but the same pathogenesis into the same disease category.

## Methods

### Subjects

Patients diagnosed with HD with proximal muscle involvement clearly described in their medical histories were included in this study. The ratio of patients with proximal HD and simple distal HD was 1:2. Patients' data were obtained from the Department of Orthopedics, Huashan Hospital, Fudan University, Shanghai, China from September 2007 to April 2022. The diagnostic criteria are presented in Table 1.

**Table 1:** The diagnostic criteria for Hirayama disease (HD).

	**Clinical manifestations**	**Imaging manifestations**	**Eletrophysiological examinations**
Elements for definite diagnosis	➀Occult onset during puberty, more common in males	➃Atrophy or thinning of the middle and lower cervical spinal cord on either neutral or flexion MRI	➅Neurogenic lesions located in anterior horns and/or roots of the middle and lower cervical spinal cord
	➁Localized muscular atrophy and weakness of the upper extremities, predominantly in the ulnar forearms and the intrinsic muscles of the hands unilaterally or mainly on one side	➄LOA or the presence of a crescent-shaped high-intensity mass at the posterior epidural space on T2WI	➆Normal or only mild abnormal conduction velocity in peripheral nerves of the upper limbs
	➂Absence of cranial nerve involvement and muscular atrophy in other parts of the body such as the lower limbs		➇Absence of obvious involvement of the cranial nerves and the thoracic, lumbar or sacral spinal cord

The proximal group included patients with HD with proximal involvement who met the following criteria: (1) definite atrophy of deltoid and/or biceps brachii; (2) decreased muscle strength in the motion of shoulder abduction and elbow bending according to the Medical Research Council grading, as determined in the medical records; (3) with or without weakness and/or amyotrophy of the supraspinatus, infraspinatus, teres major, triceps, and muscles of the forearm and the hand. Patients with HD with isolated distal involvement included those who were (1) only affected in the muscles of the hand and the forearm, and (2) did not have any weakness or amyotrophy of the supraspinatus, infraspinatus, teres major, deltoid, and biceps brachii.

### Collected data

We recorded baseline data, clinical manifestations, conventional sagittal cervical radiograms, neutral-position and cervical-flexion MRI, and electromyography (EMG) from all subjects. Baseline data included gender, age of onset, and course of illness. Clinical manifestations included affected side(s), deep tendon reflex, involvement of biceps and/or triceps, knee reflex [recorded as absent (0), decreased (+), normal (++), brisk (+++), and hyperactive (++++)], pathological reflex, and Hoffmann sign.

**Figure 1:**
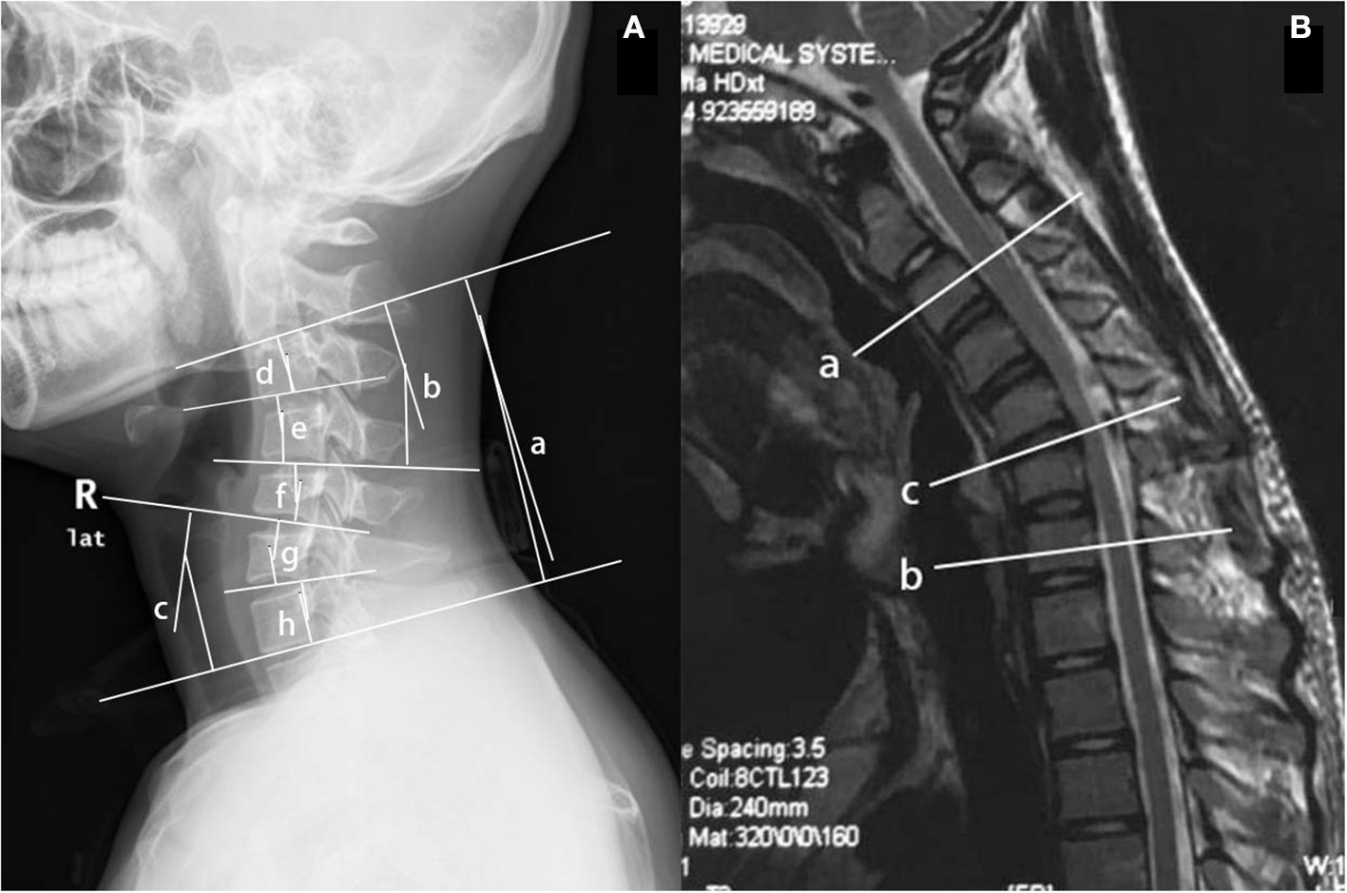
**(A)** The Cx-y Cobb angle was the angle between the two vertical lines of the two tangent lines under the inferior endplates of Cx and Cy on the conventional sagittal cervical radiograph. (a) C2-7 Cobb angle; (b) C2-4 Cobb angle; (c) C5-7 Cobb angle; (d) C2-3 Cobb angle; (e) C3-4 Cobb angle; (f) C4-5 Cobb angle; (g) C5-6 Cobb angle; and (h) C6-7 Cobb angle. **(B)** The affected segments and the most serious one on the sagittal cervical-flexion MRI. The upper end of the affected segments (a), the lower end (b), and the most serious segments (c) were recorded in the form of level of vertebral or intervertebral bodies corresponding to spinal cord segments.

Conventional sagittal cervical radiography was used to image cervical curvature (recorded as normal, loss, or kyphosis), the apex of the kyphosis, and the sagittal Cobb angle of different levels, such as C2-7, C2-4, C5-7, C2-3, C3-4, C4-5, C5-6, and C6-7. The Cx-y Cobb angle was the angle between the two vertical lines of the two tangent lines under the inferior endplates of Cx and Cy (Figure 1A). The angle was positive if the two tangents crossed behind the cervical spine, and negative if they crossed in front of the spine. The magnetic resonance imaging data included atrophy of the cervical spinal cord (from neutral-position MRI), high signal intensity on T2-weighted imaging (T2WI), segments with high signal intensity on T2WI (from cross-sectional MRI), loss of attachment (LOA) between the posterior dural sac and the subjacent lamina (presence of a crescent-shaped high-intensity mass), the whole LOA region and the most-affected segments of LOA, and the number of segments with LOA (from cervical-flexion MRI) (Figure 1B). The levels of vertebral or intervertebral bodies corresponding to spinal cord segments were recorded using cervical-flexion MRI.

Electromyography data included affected muscles and segments, and the number of segments, for waves presenting with one of the following features: positive sharp waves, fibrillation potentials, increased amplitude of motor unit potentials, or pathologic interference patterns. We analyzed different groups of muscles separately. The muscles of the hand analyzed were: first dorsal interossei, flexor pollicis longus, extensor pollicis longus, abductor pollicis brevis, extensor indicis proprius, extensor digitorum communis, and abductor digiti minimi; the muscles of the forearm were: flexor carpi radialis, flexor carpi ulnaris, and brachioradialis; the muscles of the arm were: biceps, and triceps; and the muscles of the shoulder were: deltoid, teres major, supraspinatus, and infraspinatus.

### Statistical analysis

Normally distributed quantitative variables were presented as the mean and standard deviation (SD), and were analyzed using Student's *t*-test. Qualitative data were presented as percentages and were analyzed using χ^2^-tests, adjusted χ^2^-tests, or Fisher's exact tests, as appropriate. Ordinal data were presented as the median and interquartile interval (first quartile and third quartile), and were analyzed using the signed-rank test. The repeated comparison was performed, using multivariate analysis. Statistical analysis was performed using the Statistical Product and Service Solutions (SPSS) for Windows (version 26.0; SPSS Inc., Chicago, IL, USA). The value of *p* < 0.05 was considered statistically significant.

The study was reviewed and approved by the Institutional Review Board of the authors' institute (KY2014-268), and the requirement for written informed consent was waived because of the retrospective nature of the study and the anonymous nature of the data.

## Results

### Comparison of the baseline data and clinical manifestations between the two groups

In this study, fifteen patients with HD with proximal involvement and 30 patients with simple distal HD were included (Table 2, Supplementary Table 1). All of the patients in the study were men. The ages of onset in the proximal and distal groups ranged from 16 to 25 years (17.83 ± 2.35) and 12 to 24 years (16.40 ± 2.33), respectively. The courses of illness ranged from 3 months to 8 years (2.57 ± 2.15) in the proximal group and 2 months to 6 years (2.16 ± 1.66) in the distal group, respectively. There were no statistical differences between the age of onset and the course of illness (*p* = 0.059 and *p* = 0.482).

**Table 2:** The clinical manifestations of Hirayama disease with proximal involvement.

**No**.	**1**	**2**	**3**	**4**	**5**	**6**	**7**	**8**	**9**	**10**	**11**	**12**	**13**	**14**	**15**
Age of onset/yrs	17	16	17	16	17.5	17	20	18	20	25	17	16.5	16.5	16	18
Course of illness/yrs	1	0.25	1	8	0.5	3	4	2	5	3	3	0.33	0.5	3	4
Symptom side(s)	Right	Left	Bilateral	Left	Right	Bilateral	Right	Right	Bilateral	Bilateral	Bilateral	Bilateral	Right	Right	Bilateral
**Muscle strength**															
Shoulder abduction	IV	IV	IV	IV	V	V	IV	III	V	V	V	V	IV	V	V
Elbow bend	IV	IV	IV	IV	IV	V	III	IV	IV	V	V	V	IV	V	V
Elbow extension	IV	IV	IV	IV	V	IV	IV	IV	IV	V	V	V	V	V	V
Wrist flexion	IV	IV	V	IV	V	V	IV	V	IV	V	IV	V	V	V	V
Wrist extension	IV	IV	V	IV	V	V	IV	V	IV	V	IV	V	V	V	IV
Grip	IV	IV	V	IV	V	V	III	V	IV	IV	IV	IV	IV	IV	IV
**Deep tendon reflex**															
Biceps reflex	Unknown	Unknown	+	+	+	++	+	+	+	++	++	++	+	+	+++
Triceps reflex	Unknown	Unknown	+	+	++	+	+	+	+	++	++	++	+	+	++
Knee reflex	++	+++	+++	++	+++	++++	++++	++++	++++	++++	++++	++	++	+++	+++
Hoffmann sign	Negative	Negative	Positive	Positive	Positive	Positive	Positive	Positive	Positive	Positive	Positive	Negative	Negative	Negative	Positive

*+, Decreased; ++, Normal; +++, Brisk; ++++, Hyperactive*.

In the proximal group, 3 (20.0%) patients reported symptoms on the left side, 6 (40.0%) reported symptoms on the right side, and 6 (40.0%) reported bilateral symptoms. In the distal group, 11 (36.7%), 15 (50.0%), and 4 (13.3%) patients reported left, right, and bilateral symptoms, respectively (*p* = 0.129).

After excluding missing data, the deep tendon reflexes differed significantly between the two groups. In the proximal group, 8 patients (61.5%), 4 patients (30.8%), and 1 (7.7%) patient exhibited +, ++, and +++ biceps reflexes, respectively, and 3 (11.1%), 21 (77.8%), and 3 (11.1%) patients exhibited +, ++, and +++ biceps reflexes, respectively, in the distal group (*p* = 0.005). In the proximal group, 8 (61.5%), 5 (38.5%), and 0 patients exhibited +, ++, and +++ triceps reflexes, respectively, and 4 (14.8%), 21 (77.7%), and 2 (7.5%) patients exhibited +, ++, and +++ triceps reflexes, respectively, in the distal group (*p* = 0.003). In the proximal group, 4 (26.7%), 5 (33.3%), and 6 (40%) patients exhibited ++, +++, and ++++ knee reflexes, respectively, and 15 (57.7%), 8 (30.8%), and 3 (11.5%) patients exhibited ++, +++, and ++++ knee reflexes in the distal group, respectively (*p* = 0.024).

The Hoffmann sign was positive in 10 (66.7%) patients and negative in 5 (33.3%) patients in the proximal group. The Hoffman sign was positive in 4 (13.3%) patients and negative in 26 (86.7%) patients in the simple distal group (*p* = 0.001).

### The radiological characteristics of the proximal group and the simple distal group

**Figure 2:**
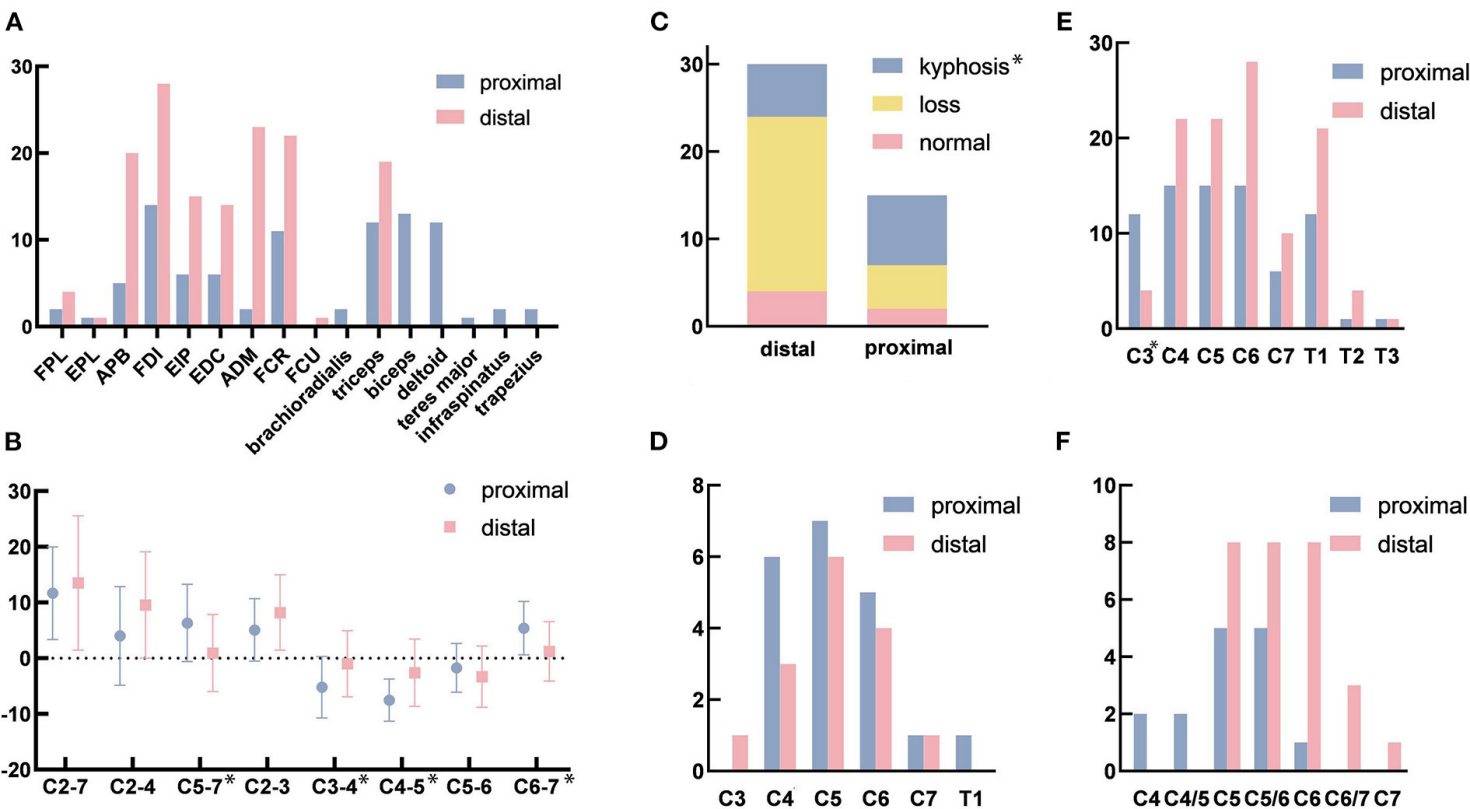
**(A)** The affected muscles in electromyography (EMG). **(B)** The sagittal Cobb angle in different levels. **(C)** The changes in the cervical curvature. **(D)** The affected segments with high signal intensity on T2-weighted imaging (T2WI). **(E)** The affected segments with the loss of attachment. **(F)** The most serious segment with the loss of attachment. FPL, flexor pollicis longus; EPL, extensor pollicis longus; APB, abductor pollicis brevis; FDI, first dorsal interossei; EIP, extensor indicis proprius; EDC, extensor digitorum communis; ADM, adductor digiti minimi; FCR, flexor carpi radialis; FCU, flexor carpi ulnaris. *p < 0.05. Figure 2 was modified from Servier Medical Art (http://smart.servier.com/), licensed under a Creative Common Attribution 3.0 Generic License (https://creativecommons.org/licenses/by/3.0/).

The cervical curvature was lordotic in 2 (13.3%) patients in the proximal group and in 4 (13.3%) patients in the simple distal group. Lordosis was lost in 5 (33.3%) patients in the proximal group and 20 (66.7%) patients in the distal group. The cervical curvature was kyphotic in 8 (53.3%) and 6 (20.0%) patients in the proximal and distal groups, respectively. The number of patients with kyphosis differed significantly between the groups (*p* = 0.039; Figure 2C). In the proximal group, the apex of the kyphosis in 3 (37.5%) patients, 4 (50.0%) patients, and 1 (12.5%) patients was located at C3, C4, and C5, respectively. In the distal group, the apex of the kyphosis was located at C4 and C5 in 3 (50.0%) patients each. The difference in the location of the apex of the kyphosis was significantly different (*p* = 0.231; Table 3).

**Table 3:** The radiological characteristics of the proximal group and the simple distal group.

**Parameters**	**Proximal**	**Distal**	***P*-value**
Kyphosis	8 (53.3%)	6 (20.0%)	0.039*
**Cobb angles**			
C2-7/°	11.67 ± 8.321	13.50 ± 12.025	0.599
C2-4/°	4.00 ± 8.840	9.57 ± 9.536	0.062*
C5-7/°	6.33 ± 6.925	0.93 ± 6.928	0.018*
C2-3/°	5.07 ± 5.599	8.20 ± 6.774	0.13
C3-4/°	−5.20 ± 5.519	−1.00 ± 5.919	0.027*
C4-5/°	−7.53 ± 3.796	−2.60 ± 6.032	0.006*
C5-6/°	−1.73 ± 4.367	−3.30 ± 5.497	0.342
C6-7/°	5.40 ± 4.793	1.23 ± 5.315	0.014*
High signal intensities on T2WI	8 (53.3%)	6 (20.0%)	0.053
**Loss of attachment**			
C3	12 (80.0%)	4 (14.3%)	0.001*
C4	15 (100%)	22 (78.6%)	0.999
C5	15 (100%)	28 (100%)	1.000
C6	15 (100%)	28 (100%)	1.000
C7	6 (40.0%)	10 (35.7%)	0.408
T1	12 (80.0%)	21 (75.0%)	0.946
T2	1 (6.7%)	4 (14.3%)	0.999
T3	1 (6.7%)	1 (3.6%)	0.999
Number of segments with loss of attachment	5.13 ± 1.25	4.21 ± 1.13	0.019*

Quantitative variables were presented as the mean and standard deviation, and qualitative data were presented as number (percentage). *P < 0.05.

The C2-7 Cobb angles were 11.67 ± 8.32 degrees in the proximal group and (13.50 ± 12.06) degrees in the simple distal group (*p* = 0.599). The C2-4 Cobb angles in the upper part of the cervical spine were (4.00 ± 8.84) degrees in the proximal group and (9.57 ± 9.54) degrees in the distal group (*p* = 0.062) The C5-7 Cobb angles in the lower part of the cervical spine were (6.33 ± 6.93) and (0.93 ± 6.93) degrees in the proximal and distal groups, respectively (*p* = 0.018). The sagittal Cobb angles in the different segments were as follows for the proximal and distal groups, respectively: C2-3 (*p* = 0.130): (5.07 ± 5.60) and (8.20 ± 6.77) degrees; C3-4 (*p* = 0.027): (−5.20 ± 5.52) and (−1.00 ± 5.92) degrees; C4-5 (*p* = 0.006): (−7.53 ± 3.80) and (−2.60 ± 6.03) degrees; C5-6 (*p* = 0.342): (−1.73 ± 4.37) and (−3.30 ± 5.50) degrees; C6-7 (*p* = 0.014): (5.40 ± 4.79) and (1.23 ± 5.32) degrees (Figure 2B, Table 3).

In the proximal group, 6 (40.0%) patients and 5 (16.7%) patients in the simple distal group exhibited cervical spinal cord atrophy (*p* = 0.140). In addition, 8 (53.3%) patients in the proximal group and 6 (20.0%) patients in the distal group showed high signal intensities in their spinal cords on T2WI (*p* = 0.053). The numbers of patients with high signal intensities on T2WI in each segment (Figure 2D) were as follows: C3 (*p* = 0.999): proximal 0 and distal 1 (3.3%); C4 (*p* = 0.999): proximal 6 (40.0%) and distal 3 (10.0%); C5 (*p* = 0.999): proximal 7 (46.7%) and distal 6 (20.0%); C6 (*p* = 0.999): proximal 5 (33.3%) and distal 4 (14.3%); C7 (*p* = 1.000): proximal 1 (6.7%) and distal 1 (3.3%); and T1 (*p* = 1.000): proximal 1 (6.7%) and distal 0 (Table 3).

The vast majority of patients with HD exhibited LOA [proximal 15 (100%) and distal 28 (93.3%)] (*p* = 0.798). The loss of attachment was observed over long segments, from C3 to T3 (Figure 2E). Among the 15 and 28 patients in each group, the numbers and the percentages of patients with LOA in each segment were as follows: C3 (*p* < 0.001): proximal 12 (80.0%) and distal 4 (14.3%); C4 (*p* = 0.999): proximal 15 (100%) and distal 22 (78.6%); C5 (*p* = 1.000): proximal 15 (100%) and distal 22 (78.6%); C6 (*p* = 1.000): proximal 15 (100%) and distal 28 (100%); C7 (*p* = 0.408): proximal 6 (40.0%) and distal 10 (35.7%), T1 (*p* = 0.946): proximal 12 (80.0%) and distal 21 (75.0%); T2 (*p* = 0.999): proximal 1 (6.7%) and distal 4 (14.3%); and T3 (*p* = 0.999): proximal 1 (6.7%) and distal 1 (3.6%). Although there was no significant difference (*p* = 0.061), the most-affected segment appeared to differ between the two groups (Figure 2F). Additionally, two patients (13.3%) in the proximal group and 0 patient in the simple distal group in both C4 vertebra-level and C4/5 intervertebral-body-level; 5 (33.3%) and 8 (28.6%) patients in both C5 vertebra-level and C5/6 intervertebral-body-level; 1 patient (6.7%) and 8 (28.6%) patients in C6; 0 and 3 (10.7%) patients in C6/7; and 0 and 1 (3.6%) patient in C7. The number of segments with LOA in the proximal group (5.13 ± 1.25) was significantly greater than that in the simple distal group (4.21 ± 1.13) (*p* = 0.019; Table 3).

### The electrophysiological characteristics of the proximal group and the simple distal group

**Figure 3:**
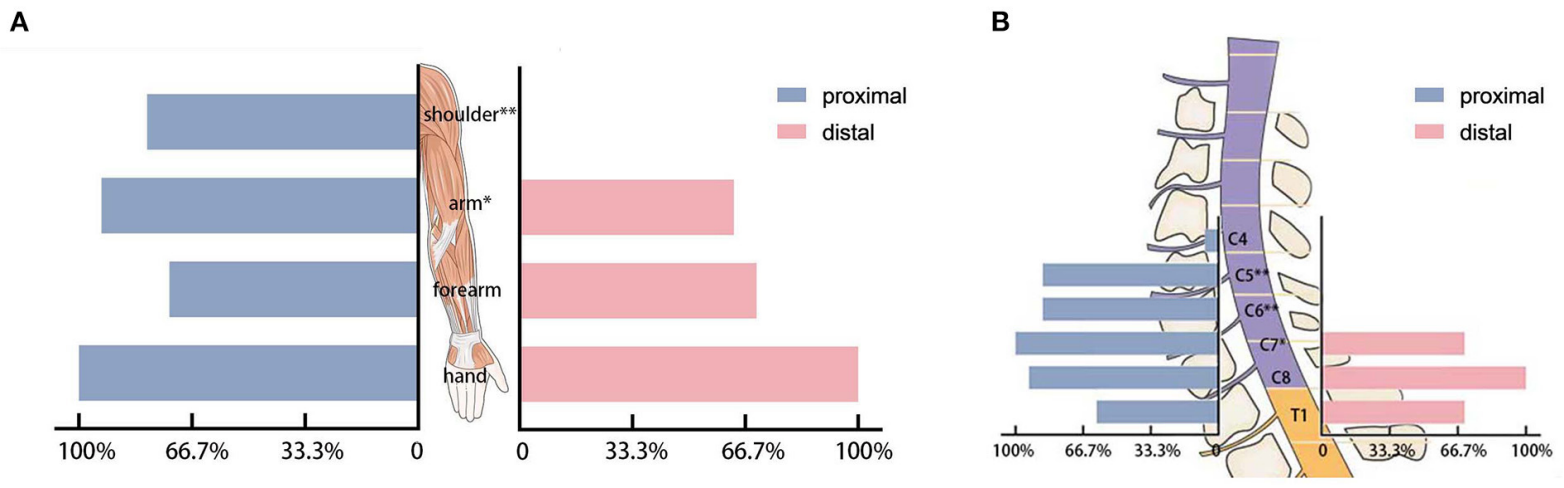
**(A)** The affected muscle groups in electromyography. **(B)** The affected cervical segments in electromyography. *p < 0.05 and **p < 0.001.

All patients with HD were affected in the muscles of the hand according to EMG. The numbers of patients affected in the muscles of the forearm were 11 (73.3%) in the proximal group and 21 (70.0%) in the distal group (*p* = 1.000). The muscles of the arm were affected in 14 (93.3%) patients in the proximal group and 19 (63.3%) patients in the distal group (*p* = 0.038). The shoulder muscles were affected in 12 (80.0%) patients in the proximal group and 0 patient in the distal group (*p* < 0.001; Figure 3A). The detailed information regarding the affected muscles is shown in Figure 2A.

In the proximal group, 1 (6.7%) patient and 0 patient in the distal group showed a neurogenic injury in the C4 segment (*p* = 0.333). In segments C5 and C6 (*p* < 0.001), 13 (86.7%) patients in the proximal group and 0 patients in the distal group showed a neurogenic injury. In segment C7 (*p* = 0.020), fifteen (100%) patients in the proximal group and 21 (70.0%) patients in the distal group showed a neurogenic injury. In segment C8 (*P* = 0.106), 13 (86.7%) patients in the proximal group and 30 (100%) patients in the distal group showed a neurogenic injury. In T1 (*P* = 0.042), 9 (60.0%) in the proximal group and 27 (90.0%) patients in the distal group had a neurogenic injury (Figure 3B). The number of affected segments in EMG in the proximal group was 4.40 ± 1.06 and the number of affected segments was 2.60 ± 0.50 in the simple distal group (*p* < 0.001).

## Discussion

Since HD was first reported in 1959 (12), it has had many names that included the word *distal*, such as *juvenile muscular atrophy of the distal upper extremity* (13), *benign juvenile muscular atrophy of the distal upper extremity* (14), *distal bimelic amyotrophy* (15), and *segmental muscular atrophy of distal upper extremity with juvenile onset* (16), which led to a stereotype that HD is a disease that only affected the distal upper limbs. However, increased understanding of the disease has led to the recognition that patients could experience amyotrophy of the proximal muscles in the upper limbs. Some studies have included patients with proximal symptoms (9, 17). Patients with proximal symptoms have been considered in the development of new diagnostic criteria and a new clinical classification system (1, 18).

The age of onset and course of illness are similar in proximal and simple distal HD, and both occur predominantly in men. The clinical manifestations were common to both types and included muscular atrophy and/or weakness of the upper extremities. The atrophied muscles in HD with proximal involvement were distributed more widely, and some patients showed generalized muscular atrophy of both upper extremities. Among patients with proximal involvement, decreased upper limb deep tendon reflex, brisk or hyperactive lower limb reflex, and positive Hoffmann sign were more common, which indicated an injury at the upper levels of the cervical spinal cord level that was more serious.

Electromyography showed that all patients were affected in the muscles of the hand. The triceps muscles, which are innervated by C7, were affected in many patients in the simple distal group according to EMG, although few patients complained of the decreased strength of elbow extension, which indicated that changes in EMG occurred prior to clinical manifestations (19). Therefore, names containing *distal* might be inappropriate, and *Hirayama disease, juvenile benign muscular atrophy of upper extremity*, and *juvenile cervical flexion myelopathy* may be better names for this disease (1). The affected segments in cases with proximal involvement extended to the rostral and/or caudal ends with C5-7 as the center, while the simple distal type primarily affected C7-T1. The number of affected segments was significantly greater in HD with proximal involvement, which indicates that the proximal type may be more serious than typical HD.

**Figure 4:**
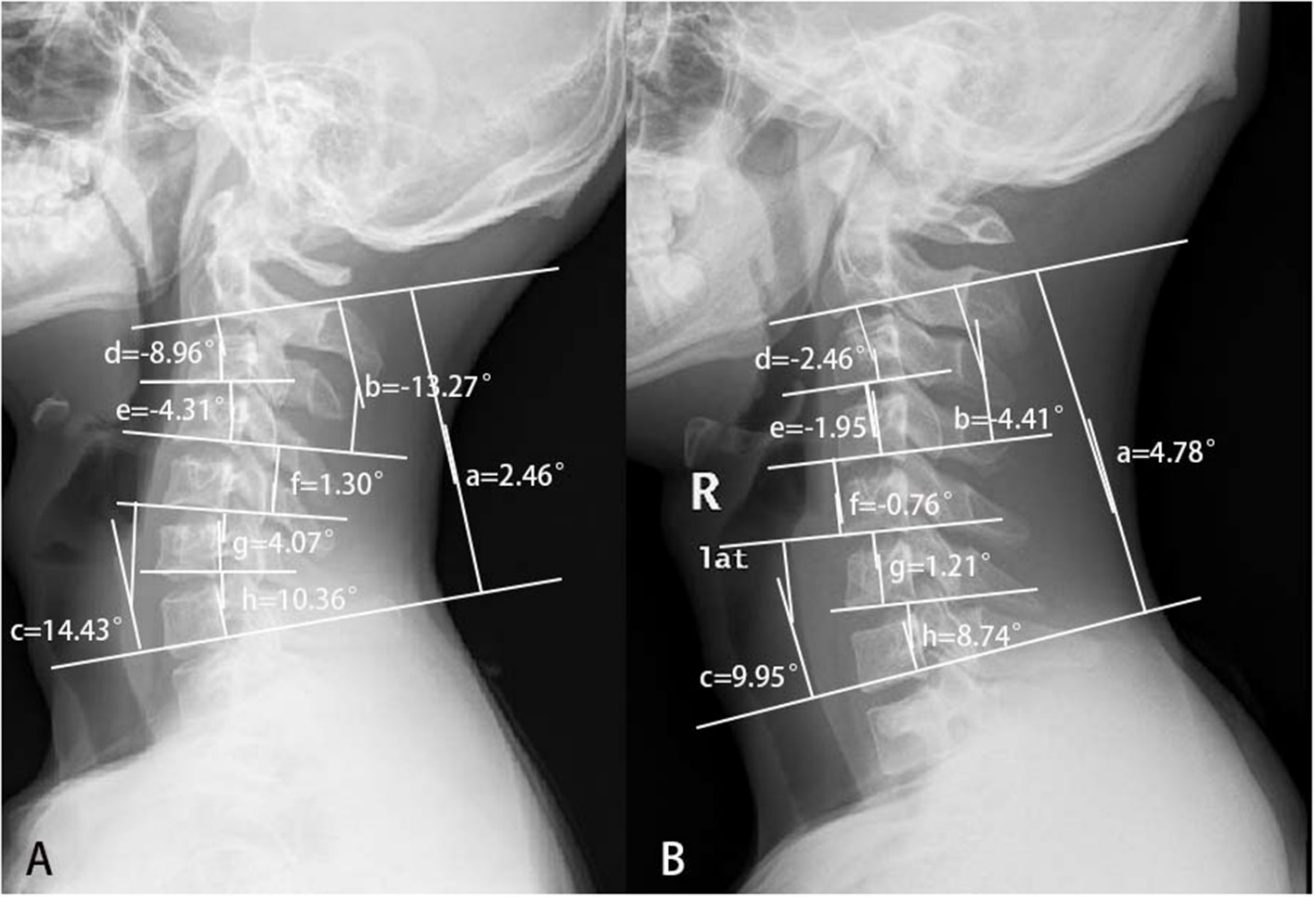
The different cervical curvatures between patients with proximal and simple distal Hirayama disease. **(A)** Hirayama disease with proximal involvement: the upper half of the cervical spine turned kyphotic, while the lower half kept lordotic. So, the whole cervical spine looked like a reverse S-shape. **(B)** Distal HD: the cervical spine turned straight. (a) C2-7 Cobb; (b) C2-4 Cobb; (c) C5-7 Cobb; (d) C2-3 Cobb; (e) C3-4 Cobb; (f) C4-5 Cobb; (g) C5-6 Cobb; and (h) C6-7 Cobb.

Conventional sagittal cervical radiography showed that the proportion of kyphosis was higher in the proximal group. Although the apex of kyphosis did not differ significantly between the two groups, the apex appeared to be higher in patients with proximal involvement. The C2-7 Cobb angle in HD was smaller than normal. The C3-4 and C4-5 showed more severe kyphosis in the proximal group than in the distal group, and C5-7 and C6-7 were straighter in the simple distal type than in the proximal type. From the mean value, divided at the level of the C4/5 intervertebral body, C2-4 was prone to kyphosis, and the lower part of the cervical spine maintained lordosis, which resulted in a reverse S-shape of the cervical spine in patients with HD with proximal involvement (Figure 4A). The cervical curvature of C5-7 was lost, resulting in a straight cervical spine in simple distal HD (Figure 4B). Such cervical curvature changes might play a part in the pathogenesis of different types of HD.

**Figure 5:**
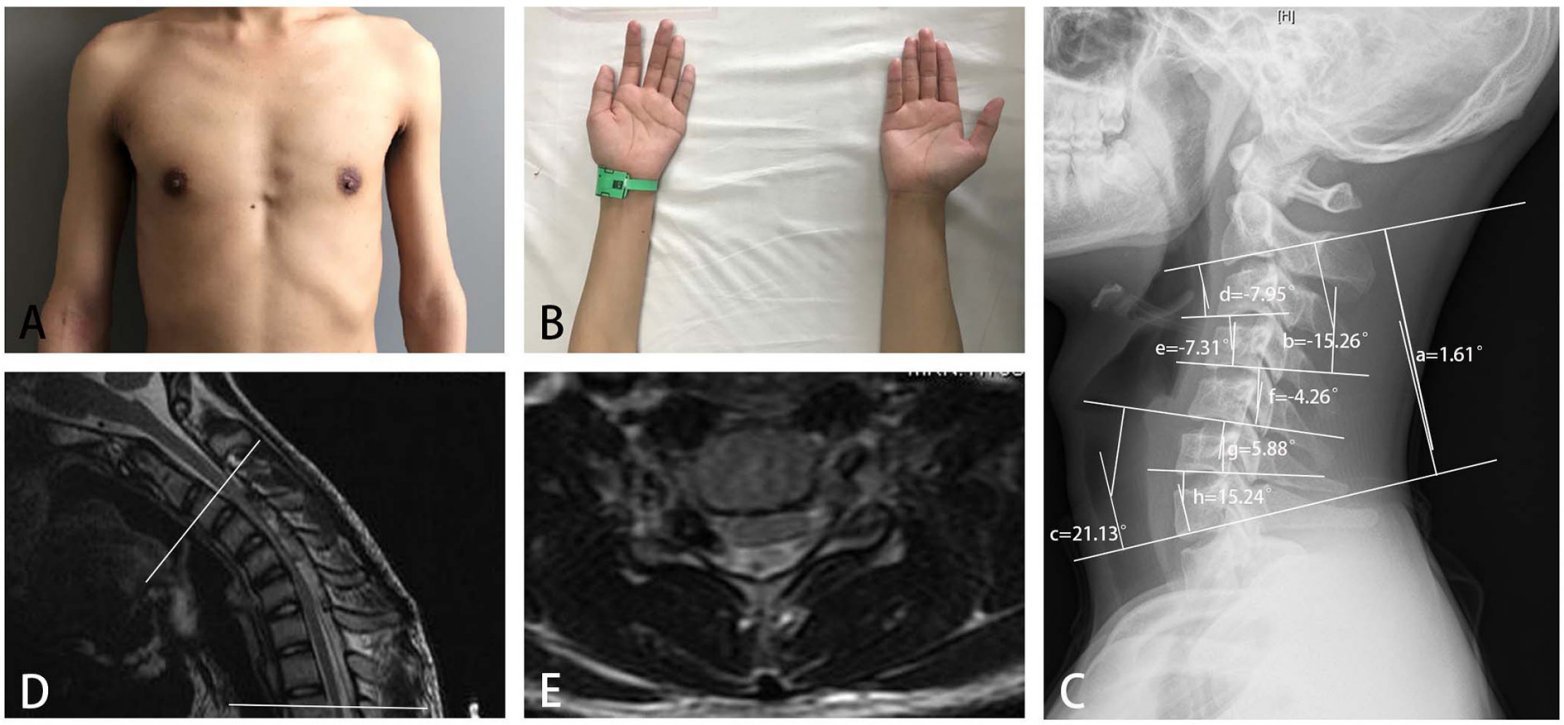
A 16-year-old boy with obvious weakness and atrophy of muscles of deltoid and biceps for 3 months **(A)**, and slight difficulty in finger extension **(B)**, was diagnosed with HD with proximal involvement. A neurogenic injury from C5 to C8 was found in electromyography. **(C)** An abnormal cervical curvature in the upper part of the cervical spine, (a) C2-7 Cobb angle = 1.61 degrees; (b) C2-4 Cobb angle = −15.26 degrees; (c) C5-7 Cobb angle = 21.13 degrees; (d) C2-3 Cobb angle = −7.95 degrees; (e) C3-4 Cobb angle = −7.31 degrees; (f) C4-5 Cobb angle = −4.26 degrees; (g) C5-6 Cobb angle = 5.88 degrees; and (h) C6-7 Cobb angle = 15.24 degrees; loss of attachment was found in cervical-flexion MRI in seven segments from C3 to T2 (between the two white lines), **(D)** sagittal plane, and **(E)** cross-sectional plane.

Cervical-flexion MRI is the most important examination for the diagnosis of HD. Among three common signs, the proportion of patients with HD with LOA was high, which indicated that LOA was useful for the diagnosis (20). The segments with LOA were longer in the proximal group than those in the distal group. In addition, LOA extended to the rostral and/or caudal ends during the progression of HD (21), so some of the affected segments were very long. Therefore, a wider range of atrophied muscles, higher proportions of brisk or hyperactive knee reflexes and positive Hoffmann sign, and more affected segments observed in EMG and MRI indicated that HD with proximal involvement (Figure 5) was likely to be more serious than typical HD, and early correct diagnosis and treatment was likely to be more urgent in patients with proximal involvement. The strength of posterior cervical extensors and cervical sagittal alignment were closely related, indicating that the weakness of posterior cervical extensors might predispose the cervical spine of patients with HD to a less stable situation (22).

A total of eighteen case reports, including 19 patients with HD with proximal involvement, were reported in English studies, as shown in Table 4 (3–5, 23–37). A number of these patients were affected in the proximal and distal muscles of the upper limb. There were some characteristics in 6 patients with HD with proximal involvement, which indicated these cases had a common pathogenesis with the simple distal type (9). Therefore, the HD with proximal involvement could not be ignored in clinical practices.

**Table 4:** Summary of proximal Hirayama disease case reports.

**First writer**	**Hiroaki**	**Andreadou**	**Andreadou**	**Catalina**	**Jung**	**Renard**	**Gowda**	**Foster**	**Sim**	**Yoo**
Age of onset/yrs	22	Unknown	39	20	16	30	18	16	20	13
Gender	Male	Male	Male	Male	Male	Male	Male	Male	Male	Male
Symptom side(s)	Right	Right	Bilateral	Right	Right	Left	Bilateral	Bilateral	Right	Left
Affected muscles	P/A/H	A/F/H	A/H	A/F/H	A	S/A	S/A/F/H	A/H	P/A/F/H	S
Upper limb deep tendon reflex	+	++	++	Not mentioned	–	++	++	++	++	+++
Lower limb deep tendon reflex	++	++	++	Not mentioned	++	++	++	++	++	+++
Pathological reflex	Negative	Negative	Negative	Not mentioned	Negative	Negative	Negative	Negative	Negative	Positive
LOA	Exist	Unknown	Unknown	Exist	Exist	Not exist	Exist	Exist	Exist	Exist
Denervation in EMG	Exist	Exist	Exist	Exist	Exist	Exist	Exist	Exist	Exist	Exist
Nationality	Japan	Cyprus	Cyprus	America	Korea	France	India	Italy	Australia	Korea
Year of publishing	2006	2009	2009	2011	2012	2012	2013	2013	2013	2015
**First writer**	**Holla**	**Lee**	**Kim**	**Brambilla**	**Al-Ghawi**	**Takahito**	**Yokote**	**Wu**	**Narra**
Age of onset/yrs	19	18	20	45	24	12	<18	28	17
Gender	Male	Male	Male	Male	Male	Female	Male	Female	Male
Symptom side(s)	Bilateral	Right	Left	Right	Left	Right	Bilateral	Bilateral	Right
Affected muscles	P/B/S/A/F/H	S/A	S/A/F/H	P/B/S/A/F/H	S/A	S/A/F/H	S/A	A/F/H	A/F
Upper limb deep tendon reflex	++	++	++	++	++	+	+	+	Not mentioned
Lower limb deep tendon reflex	++	++	++	++	++	+++	Not mentioned	+++	Not mentioned
Pathological reflex	Negative	Negative	Negative	Negative	Negative	Positive	Not mentioned	Positive	Not mentioned
LOA	Exist	Exist	Not exist	Not exist	Unknown	Exist	Exist	Exist	Exist
Denervation in EMG	Exist	Not mentioned	Exist	Exist	Exist	Exist	Exist	Not mentioned	Exist
Nationality	India	Korea	Korea	Italy	Bahrain	Japan	Japan	China	India
Year of publishing	2015	2016	2016	2016	2016	2017	2019	2019	2021

*A, muscles of arm; B, muscles of back; F, muscles of forearm; H, muscles of hand; LOA, loss of attachment; P, pectoralis; S, muscles of shoulder. +, Decreased; ++, Normal; +++, Brisk*.

For HD with proximal involvement, amyotrophy appeared in both the proximal and distal upper extremities in many patients, and may be bilateral. Therefore, a clear differential diagnosis of some neurological diseases, such as motor neuron disease, is critical. Diagnosis of HD with proximal involvement requires greater caution, and treatment approaches should be more conservative. Patients should wear a cervical collar and followed-up closely after 4–6 months. Other treatments could not be chosen until other neurological diseases had been excluded.

Our study was subject to several limitations. First, the small sample size limited the interpretation of the conclusions. However, this was the largest study to date that focused on HD with proximal involvement. In addition, this study included only one center. Multicenter studies are expected to be performed in the future. Finally, this was a retrospective study, which might have resulted in bias due to incomplete clinical data, and heterogeneous data sources for physical examinations and medical records performed by different clinicians.

## Conclusion

Hirayama disease with proximal involvement is a more serious condition than typical HD. Loss of cervical curvature in the upper cervical spine was more severe in cases with proximal involvement, and the affected segments in MRI and EMG were longer in HD with proximal involvement. The diagnosis of HD with proximal involvement should be approached with caution and with the goal of maximizing precision.

## Data availability statement

The original contributions presented in the study are included in the article/Supplementary material, further inquiries can be directed to the corresponding authors.

## Ethics statement

The study was reviewed and approved by the Institutional Review Board of Fudan University (KY2014-268), and the requirement for written informed consent was waived because of the retrospective nature of the study and the anonymous nature of the data. Written informed consent was obtained from the individual(s) for the publication of any potentially identifiable images or data included in this article.

## Author contributions

HongwW: conceptualization, methodology, software, formal analysis, resources, and writing the original draft. YT and JW: software and formal analysis. CS, CN, CZ, FZ, XX, XM, and FL: writing, reviewing, and editing the article and supervision. JJ: writing, reviewing and editing the article, supervision, and funding acquisition. HonglW: conceptualization, methodology, writing, reviewing and editing the article, supervision, and funding acquisition. All authors contributed to the article and approved the submitted version.

## Funding

This study was funded by the Clinical Research Plan of SHDC (Grant No. SHDC2020CR4030), the Clinical Technology Innovation Project of SHDC (Grant No. SHDC12019X26), the National Natural Science Foundation of China (Grant No. 82072488), and the AO Spine National Research Grant 2020 [No. AOSCN(R)2020-9].

## Conflict of interest

The authors declare that the research was conducted in the absence of any commercial or financial relationships that could be construed as a potential conflict of interest.

## Publisher's note

All claims expressed in this article are solely those of the authors and do not necessarily represent those of their affiliated organizations, or those of the publisher, the editors and the reviewers. Any product that may be evaluated in this article, or claim that may be made by its manufacturer, is not guaranteed or endorsed by the publisher.
