# Supplementary material for: The radiological and electrophysiological characteristics of Hirayama disease with proximal involvement: A retrospective study
Source: Front Neurol. 2022 Aug 11;13:969484. doi: 10.3389/fneur.2022.969484 (PMC9406812; doi:10.3389/fneur.2022.969484)
Supplement: Supplementary file 1 [file Table_1.pdf]

Supplementary Table 1: The Clinical Manifestations of the Simple Distal Hirayama Disease

No.	**1**	**2**	**3**	**4**	**5**	**6**	**7**	**8**	**9**	**10**	**11**	**12**	**13**	**14**	**15**
Age of onset/yrs	14.5	18	14.5	15	15	15	24	14.5	12	16.5	17.5	16.5	18	17.5	16
Course of illness/yrs	0.5	3	2.5	1	0.67	2	2	2.5	5	2.5	1	0.25	1	4.5	1
Symptom side(s)	Right	Bilateral	Left	Left	Right	Right	Left	Left	Right	Left	Bilateral	Right	Right	Left	Left
Muscle strength															
Shoulder abduction	V	V	V	V	V	V	V	V	V	V	V	V	V	V	V
Elbow bend	Unknown	Unknown	V	V	V	V	V	V	V	V	V	V	V	V	V
Elbow extension	Unknown	Unknown	V	V	V	V	V	V	V	V	V	V	V	V	V
Wrist flexion	Unknown	Unknown	V	V	V	V	V	V	V	V	V	V	V	V	V
Wrist extension	Unknown	Unknown	IV	V	V	V	V	V	V	V	V	V	V	V	V
Grip	Unknown	Unknown	V	IV	IV	IV	IV	IV	IV	IV	IV	IV	IV	IV	IV
Deep tendon reflex															
Biceps reflex	++	++	++	++	++	++	++	++	+	++	++	++	+	++	++
Triceps reflex	++	++	++	+	++	++	++	++	+	++	++	++	+	+++	++
Knee reflex	++	++	+++	Unknown	++	++	+++	++	++	+++	++	+++	+++	+++	++

Hoffmann sign	-	-	-	-	-	-	+	-	-	-	-	-	-	-	-

+: Decreased; ++:Normal; +++: Brisk; ++++:Hyperactive.

No.	16	17	18	19	20	21	22	23	24	25	26	27	28	29	30
Age of onset/yrs	17	19.5	16	18	15.5	15	15	18	15	15.5	20.5	16.5	13	18	15
Course of illness/yrs	0.17	5.5	3	5	1.5	2	1	6	4	1.5	0.25	1.5	1	2	1
Symptom side(s)	Right	Left	Right	Right	Left	Right	Right	Left	Right	Right	Right	Left	Bilateral	Right	Left
Muscle strength															
Shoulder abduction	V	V	V	V	V	V	V	V	V	V	V	V	V	V	V
Elbow bend	V	V	V	V	V	V	V	V	V	V	V	V	V	V	V
Elbow extension	V	V	V	V	V	V	V	V	V	V	V	V	V	V	III
Wrist flexion	V	V	V	IV	IV	V	V	V	V	IV	V	IV	III	III	II
Wrist extension	IV	IV	IV	IV	IV	V	V	V	V	V	IV	IV	III	II	III
Grip	IV1	IV	IV	IV	IV	III	III	III	III	III	III	III	III	III	II
Deep tendon reflex															
Biceps reflex	Unknown	Unknown	Unknown	++	++	++	++	++	++	++	+++	+++	+	++	+++
Triceps reflex	Unknown	Unknown	Unknown	++	++	++	++	++	++	++	+++	++	+	++	++
Knee reflex	Unknown	Unknown	Unknown	++	++	++++	++	+++	++	++	++++	+++	++++	++	++
Hoffmann sign	-	-	-	-	-	-	-	-	-	-	+	-	+	-	-
